# Identifying the Antiproliferative Effect of *Astragalus* Polysaccharides on Breast Cancer: Coupling Network Pharmacology With Targetable Screening From the Cancer Genome Atlas

**DOI:** 10.3389/fonc.2019.00368

**Published:** 2019-05-17

**Authors:** Cun Liu, Huayao Li, Kejia Wang, Jing Zhuang, Fuhao Chu, Chundi Gao, Lijuan Liu, Fubin Feng, Chao Zhou, Wenfeng Zhang, Changgang Sun

**Affiliations:** ^1^First School of Clinical Medicine, Shandong University of Traditional Chinese Medicine, Jinan, China; ^2^College of Basic Medical, Shandong University of Traditional Chinese Medicine, Jinan, China; ^3^Department of Basic Medical Sciences, School of Medicine, Xiamen University, Xiamen, Fujian, China; ^4^Department of Oncology, Weifang Traditional Chinese Hospital, Weifang, China; ^5^School of Traditional Chinese Medicine, Beijing University of Chinese Medicine, Beijing, China; ^6^School of Clinical Medicine, Weifang Medical University, Weifang, China; ^7^Department of Oncology, Affiliated Hospital of Shandong University of Traditional Chinese Medicine, Weifang, China

**Keywords:** *Astragalus* polysaccharide, breast cancer, network pharmacology, proliferation inhibition, TCGA

## Abstract

**Background:** *Astragalus* polysaccharides (APS), natural plant compounds, have recently emerged as a promising strategy for cancer treatment, but little is known concerning their effects on breast cancer (BC) tumorigenesis.

**Methods:** We obtained breast cancer genetic data from The Cancer Genome Atlas (TCGA) database, network pharmacology to further clarify its biological properties. Survival analysis and molecular docking techniques were implemented for the final screening to obtain key target information. Our experiments focused on the detection of intervention effects of APS on BC cells (MCF-7 and MDA-MB-231), and quantitative RT-PCR (qRT-PCR) was used to assess the expression of key targets.

**Results:** A total of 1,439 differentially expressed genes (DEGs) were identified by TCGA and used to build disease networks. Module analysis, gene ontology and pathway analysis revealed characteristic of the DEGs network. Topological properties were used to identify key targets, survival analysis and molecular docking finally found that the targets of APS regulation of BC cells may be CCNB1, CDC6, and p53. Through cell viability, migration and invasion assays, we found that APS interferes with the development of breast cancer in MCF7 and MDA-MB-231 cells in a dose-dependent manner. Furthermore, qRT-PCR verification suggested that the expression of CCNB1 and CDC6 in breast cancer cells was significantly downregulated in response to APS, while expression of the tumor suppressor gene P53 was significantly increased.

**Conclusion:** Results of this study suggest therapeutic potential for APS in BC treatment, possibly through interventions with CCNB1, CDC6, and P53. Furthermore, these findings illustrate the feasibility of using network pharmacology to connect large-scale target data as a way to discover the mechanism of natural products interfering with disease.

## Introduction

Breast cancer (BC) is one of the most common cancers and remains a serious health threat for women, comprising ~30% of cancer cases in women each year (1). Although survival rates of breast cancer patients have gradually improved thanks to new therapeutic strategies, many patients still face recurrence, and long-term mortality remains high. In addition, many treatments are accompanied by drug resistance and serious side effects, which affect patients' quality of life (2, 3). Therefore, it is necessary to identify new therapeutic agents or emerging targets.

Small molecular compounds from natural products have historically been used for disease intervention, and as valuable sources of lead compounds for drug development. Accumulating knowledge suggests that many diseases manifest as complex systems, which do not seem able to effectively respond to a specific, single treatment (4, 5). The multi-target interventional properties of natural products seem to fit this therapeutic concept. With the continuous development of natural products as effective candidates for drug selection, comprehensive determination of small molecule multi-target interaction spectrums have become increasingly necessary. However, the lack of a complete pharmacological understanding of drug function mechanisms has hampered the wider application of natural products in drug development.

Network pharmacology provides a system-level approach to revealing potentially complex relationships between multiple components and multiple targets (6). The principle of network intervention is especially applicable to the treatment of tumors. Clinically efficacious cancer treatments are usually multi-targeted, as the effects of oncogenes are known to be multi-genic, and this joint method aims to discover unknown targets for existing drugs (7). Of course, this also depends to some extent on the continuous development of bio-big data to provide original material.

The Cancer Genome Atlas (TCGA) database provides these accumulated raw materials. The TCGA network contains a molecular atlas of tumors from 11,160 patients across 33 cancer types, which aims to catalog and discover major oncogenic genome alterations and to create a comprehensive “landscape” of cancer genomic profiles (8). Many achievements using these data have already been published, involving cancer diagnosis, treatment and prevention (9–11). Thorough TCGA molecular data has led to a significant increase in our knowledge of cancer biology, and its availability has provided an unprecedented opportunity to expand understanding of tumor mechanisms (12, 13). At the same time, analyses of TCGA data are usually complex. Choosing the appropriate computational analysis methods determines whether we can obtain improved biological and medical insights.

Here, in order to clarify the comprehensive mechanisms of *Astragalus* polysaccharides (APS), we adopted a systematic approach based on network pharmacology to screen out the network targets and functional characteristics of APS intervention in breast cancer. *In vitro* experiments were used to further verify the validity of the results. Based on the target-sets and dominant network properties, network pharmacology provides a feasible way of thinking about natural products to interfere with complex disease.

## Materials and Methods

### Data Source and Processing

DNA expression data for BC were downloaded from the TCGA database (https://cancergenome.nih.gov/) (14). DNA expression data for 1,208 samples were obtained, including 112 normal samples and 1,096 BC samples. These samples have complete survival data and are histologically typed as BC. Since the information was retrieved from TCGA database, a public data set, further ethical approval was not needed for our research. Data collection and processing procedures were in accordance with TCGA data access and policies for protecting human subjects (http://cancergenome.nih.gov/publications/publicationsguidelines). Subsequently, based on the edgeR software package in the R platform, downloaded DNA data were normalized and analyzed for differences to identify differentially expressed genes.

### DEGs Analysis and Network Construction

The Database fonotar Antion, Visualization and Integrated Discovery (DAVID: https://david.ncifcrf.gov/) (15), a comprehensive set of functional annotation tools, was utilized for GO and KEGG analysis of the DEGs. When we ran DAVID analyses, the cut-off threshold of the display path was selected as *p* < 0.05.

String database (https://string-db.org/cgi/input.pl) and Cytoscape 3.5.1 software were employed to construct a protein-protein interaction (PPI) network for invasive breast cancer DEGs (16). CytoNCA and the MCODE plug-in were used to perform a topological analysis and module analysis of the PPI network, respectively. The CytoNCA plug-in performs topology analysis based on “betweenness (BC),” “closeness (CC),” “degree (DC),” “eigenvector (EC),” “local average connectivity-based method (LC),” “network (NC),” “subgraph (SC),” and “information (IC)” (17). The plug-in MCODE was used with the default parameters (degree cut-off ≥ 2, node score cut-off ≥ 0.2, K-core ≥ 2, and max depth = 100) (18). Subsequently, based on these two analyses, the main nodes in the PPI network were identified comprising the main DEGS that can be used as biomarkers of BC.

### Kaplan-Meier Test

Kaplan-Meier Plotter (http://kmplot.com/analysis/), an online tool, was used to assess the effect of 54,675 genes on survival using 10,461 cancer samples (5,143 breast, 1,816 ovarian, 2,437 lung, and 1,065 gastric cancer) and to perform survival analysis on DEGs (19). Recurrence-free survival refers to the recurrence-free survival of BC patients, and the overall survival (OS) is the time from diagnosis to any cause of death. Kaplan-Meier Plotter's chi-square test was used to identify the relationship between DEGs and BC survival (20). When the *P*-value of RFS and OS were <0.05, it is considered that DEGs are associated with the survival of BC.

### Molecular Docking

The Surflex-Dock program interfaced with Sybyl X.0 was utilized to dock APS with DEGs (21). The structure of APS was constructed using ChemDraw Ultra 12.0 and optimized in Sybyl X (22). The X-ray crystal structure of DEGs was extracted from the RCSB protein database (http://www.rcsb.org/), and the co-crystal ligands and structural water molecules were removed from the crystal structures before the docking simulation began. A polar hydrogen atom was added, and the Kollman-total atomic charge was assigned to the protein atom. Surflex-Dock is a fully automated and flexible docking program for ligands that relies on the rigid-receptor approximation to simulate ligand-receptor binding mode (23). In our study, the ligand model was based on considering the structural similarity of the co-crystallized ligand and target compound, setting the ProtoMol expansion and ProtoMol threshold parameters to default values of 0 and 0.50 (24), respectively, creating a binding pocket for the study. Key DEGs were identified as those that could dock with APS.

### Gene Set Enrichment Analysis (GSEA) and Characterization of Key Targets

We further used GSEA to analyze the association between key targets and potential biological mechanisms. A single gene was used as a phenotype, the degree of ranking key mRNAs was calculated based on Pearson correlation coefficient. Expression on the box plot was used to characterize difference expression of key targets in the sample. |Log2FC| Cutoff and *p*-value Cutoff were set to 1 and 0.01, respectively. The cBioPortal database (http://www.cbioportal.org) shows the co-mutation gene of CCNB1 and CDC6, respectively.

### Effect of APS on Cellular Viability

The hormone receptor positive breast cancer cells (MCF-7) and triple negative breast cancer cells (MDA-MB-231) were seeded in the 96-well culture plates at a density of 1 × 10^3^ cells/well and incubated for 24 h at 37°C and 5% CO_2_ atmosphere, respectively. Cells were exposed to 0, 0.25, 0.5, 0.75, 1, and 2 mg/mL APS (98% purity, purchased from Shanghai Yingxin Co., Ltd.) and further incubated for 0, 24, 48, 72, and 96, respectively. The medium was replaced with 100 μL of fresh medium containing 10% CCK8 (WST-8, yiyuanbiotech) (25), and cells were incubated for 4 h at 37°C and 5% CO_2_ atmosphere. The OD450 nm absorbance value in each well was determined by the scanning porous spectrophotometer (Thermo Scientific, China). Cell proliferation rate (%) was used to describe the effect of APS on cellular viability, and calculated as follow equation:

$$\begin{array}{r} \text{Cellproliferationrate}\left(\%\right)=\left(\text{ODAPS }-\text{ ODBlank}\right)/\\ \left(\text{ODControl }-\text{ ODBlank}\right)\times \;100\% \end{array}$$

### Cell Migration Assay

After adjusted concentration to 1 × 10^5^ cells/mL with complete medium, the cells were seeded in a 6-well culture plate, the back of which had been marked with 0.5–1 cm horizontal line across a hole, and incubated overnight at 37°C and 5% CO_2_ atmosphere to attach as a monolayer of cells. The horizontal lines were scratched using a 1 mL pipette tip as far as possible perpendicular to the back, and then the isolated well cells were washed three times and removed with warm PBS. The cells were exposed to 0, 0.25, and 0.5 mg/mL APS for 24 h with serum-free DMEM medium at 37°C and 5% CO_2_ atmosphere. The distance of cell movement was measured and photographed under an inverted microscope (100×). The scratch assay was performed at least 5 times, and five fields were selected randomly every time.

### Transwell Invasion Assay

After adjusted at the concentration of 1 × 10^5^ cells/mL with the serum-free medium, The MDA-MB-231 cells were seeded 100 μL/well into the upper chamber of the transwell migration chamber. The medium with a concentration of 0, 0.25, 0.5 mg/mL of APS was added lower chamber. The cells were then incubated for 24 h at 37°C and 5% CO_2_ atmosphere. The chamber was removed and fixed with methanol for 20 min. After dried at room temperature, the cells were stained with crystal violet for 20 min. Cells that did not pass through the upper part of the chamber were removed with a wet cotton swab, and then the chamber was placed under an inverted microscope to calculate remaining cells.

### Quantitative RT-PCR of CCNB1, CDC6, and P53

After adjusted concentration to 1 × 10^5^ cells/mL with complete medium, the MDA-MB-231 cells were seeded 6.0 mL into the cell culture dish (*d* = 100 mm), and incubated 24 h at 37°C and 5% CO_2_ atmosphere. And then the cells were exposed to 0, 0.25 and 0.5 mg/mL of APS, and incubated for 24 or 48 h at 37°C and 5% CO_2_ atmosphere, respectively. The total RNA of MDA-MB-231 cells was extracted with qRT-PCR Trizol reagent (Vazyme, Nanjing, Jiangsu, China). And the cDNA was synthesized by reverse transcriptionusing a Transciptor First Strand cDNA Synthesis Kit (Vazyme). To prepare template DNA, the amplification reaction was as follows: 3 min at 95°C, followed by 40 cycles. Two duplicate systems were used for each group. Primer sequences were shown as follows: 5′-GCGTGTGCCTGTGACAGTTA-3′ (forward) and 5′-CCTAGCGTTTTTGCTTCCCTT-3′ (reverse) for CCNB1; 5′-ATGGCGCGTTCTCTGCTCACTACA-3′ (forward) and 5′-CTCACGCCTATAATCCCAGCACTT-3′ (reverse) for CDC6; 5′-GGCCATCTACAAGCAGTCACAG-3′ (forward) and 5′-AGCAAATCTACAAGCAGTCACAG-3′ (reverse) for P53.

### Statistical Analysis

Quantitative analysis of data were performed using GraphPad Prism 6.0 statistical software (GraphPad Software Inc., La Jolla, CA) and Image J software (National Institutes of Health, USA). Data were expressed as the mean ± sd. The difference among groups was determined by one-way ANOVA tests and Kruskal-Wallis tests using SPSS version 12.0 (IBM SPSS Statistics, Chicago, IL, USA). Differences were considered significant if *P* < 0.05.

## Results

### Identification of Differentially Expressed Genes

**Figure 1:**
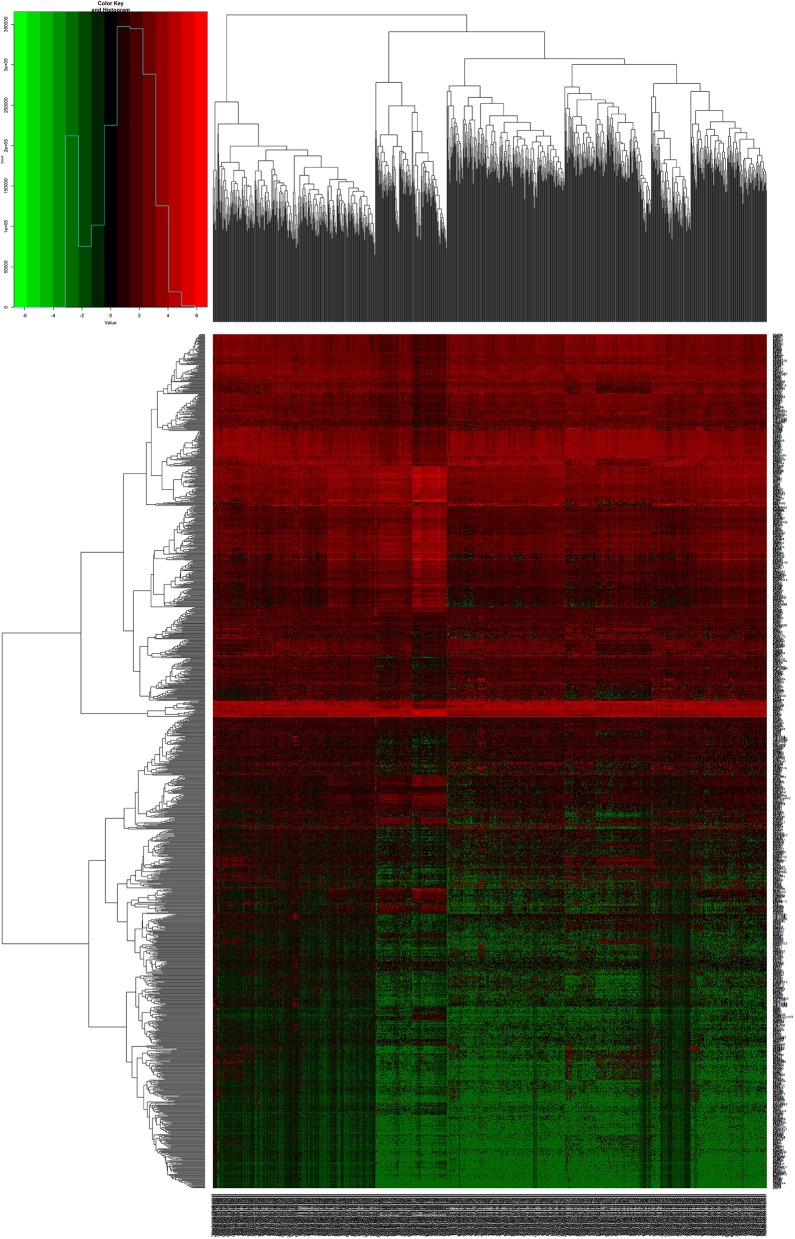
Heat maps of breast cancer-related differentially expressed DEGs. The color from green to red shows a trend from low expression to high expression.

**Figure 2:**
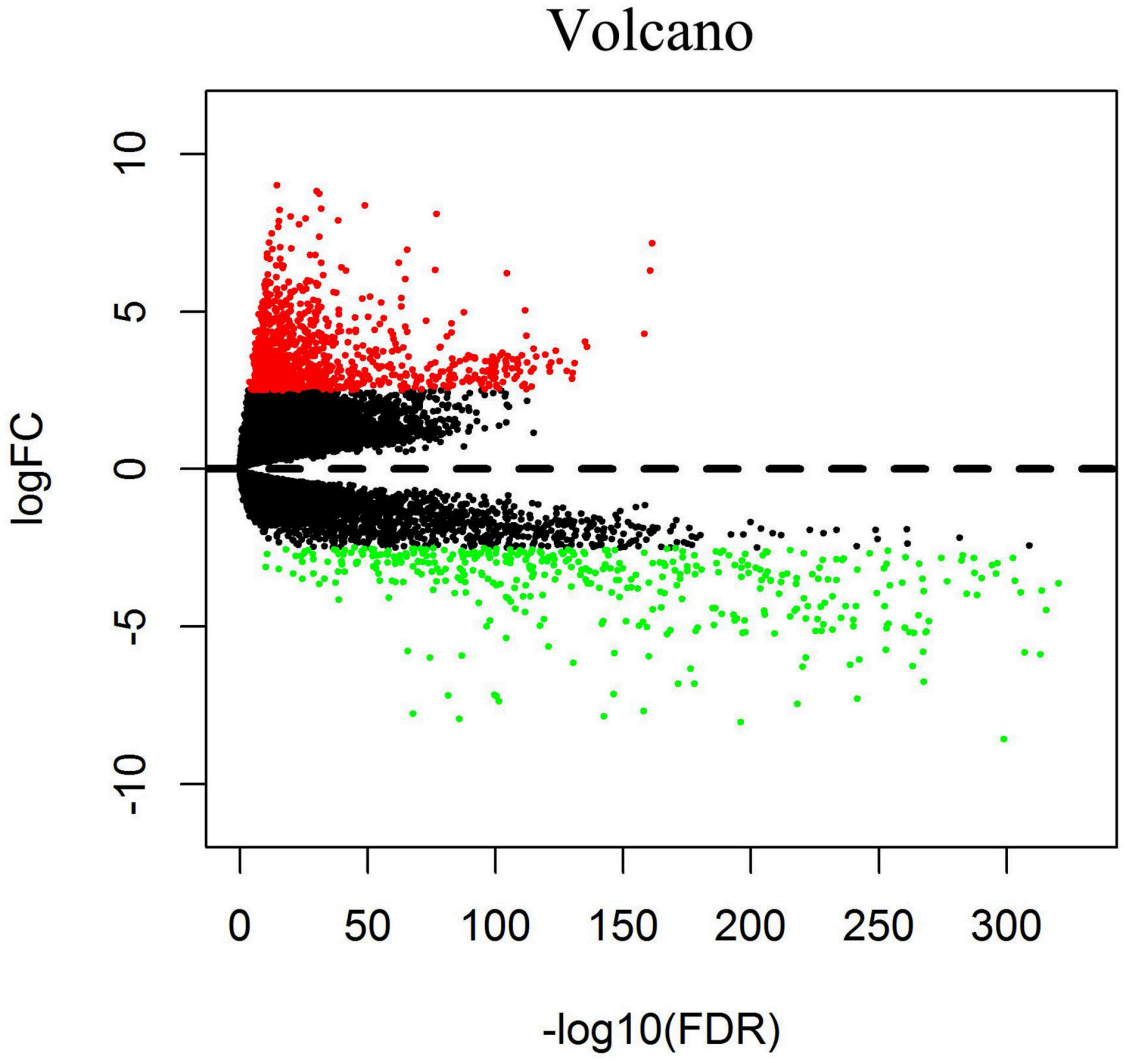
Volcano diagrams of BC-related differentially expressed DEGs. Red dots represent upregulated DEGs, and green dots represent downregulated DEGs.

We downloaded 1,208 DNA expression datasets and identified DEGs by calculating the difference in gene expression between 112 normal samples and 1,096 BC samples. Using the LIMMA software package, differentially expressed data were extracted and analyzed. Using | log 2-fold change | ≥ 2.5 and *P*-value <0.01 as screening cutoff conditions, 1,439 DEGs were screened, containing 957 upregulated genes and 482 downregulated genes (Figures 1,2).

### DEGs Enrichment Analysis and PPI Network Analysis

**Figure 3:**
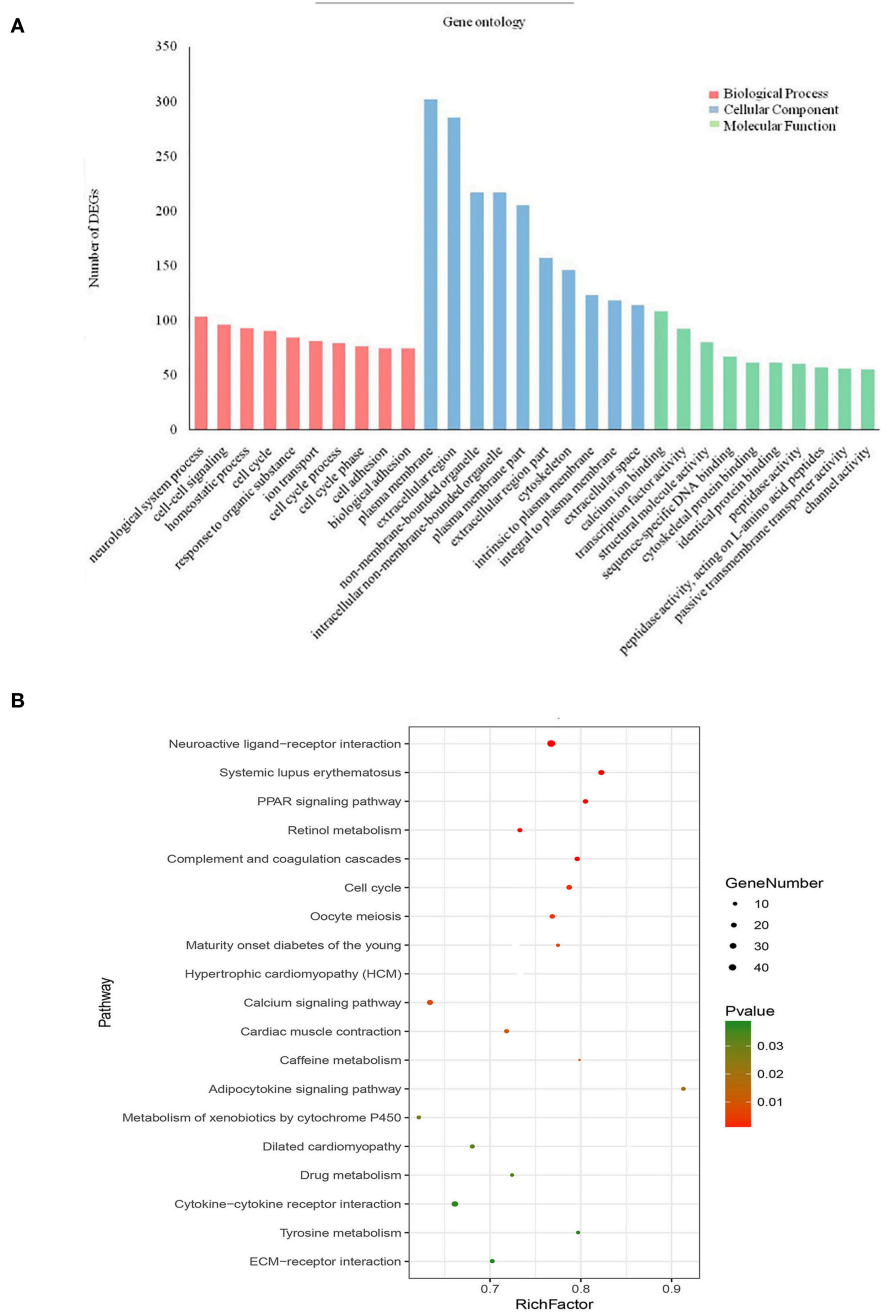
Enrichment of gene ontology terms and KEGG pathways for DEGs. **(A)** Biological process, Cellular component and Molecular function for DEGs (*P* < 0.05). **(B)** KEGG pathways for DEGs (*P* < 0.05).

To further analyse DEGs in BC, we used the DAVID database to perform GO analysis and pathway enrichment analysis on DEGs. GO analysis, including molecular function, biological process and cellular component (Figure 3A), was performed on 1,439 DEGs, with *p* < 0.05 as the cut-off criterion. Enrichment results show that: Molecular function participates in cellular activities, such as calcium ion binding, transcription factor activity, sequence-specific DNA binding, cytoskeletal protein binding and peptidase activity; Biological process is mainly enriched in cell-cell signaling, cell cycle, ion transport, and cell adhesion and other physiological processes related to cell growth, division, and proliferation; Cellular component is associated with the extracellular space, plasma membrane binding, extracellular regions, and components of the plasma membrane.

KEGG pathway enrichment analysis of the 1,439 DEGs was also performed in DAVID, for which the cut-off criterion was *P* < 0.05. Pathway enrichment shows that the biological processes involved in these DEGs are mainly neuroactive ligand-receptor interaction, cytokine-cytokine receptor interaction, calcium signaling pathway, and cell cycle signaling pathway (Figure 3B).

**Figure 4:**
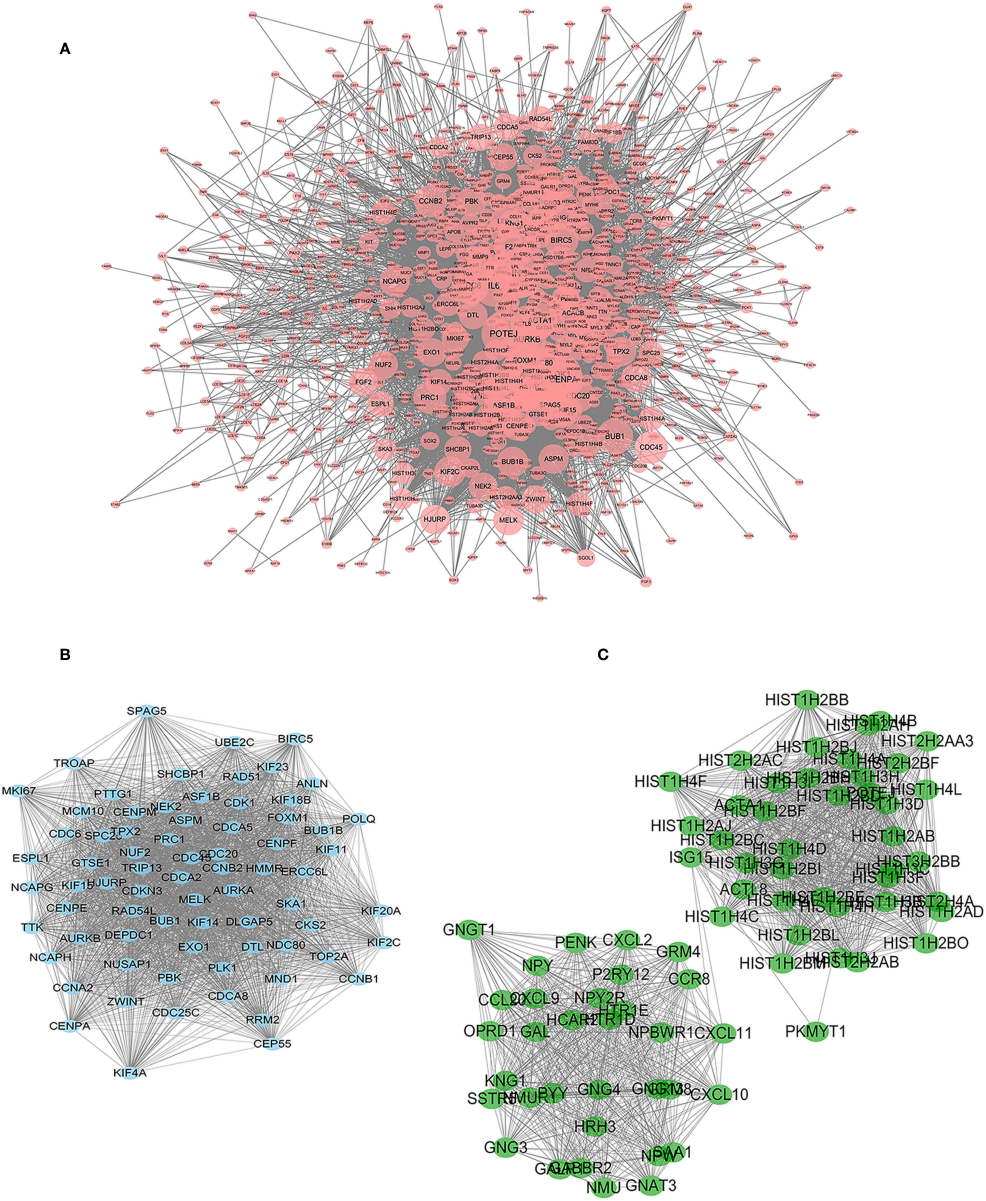
PPI network of significant DEGs. Two subnetworks and 148 nodes were identified by Cytoscape MCODE plugin. **(A)** PPI network with 1,133 nodes and 11,112 edges. The size of the node increases with the size of the degree. **(B)** Module 1 contains 73 nodes and 2,497 edges (*P* < 0.05). **(C)** Module 2 contains 75 nodes and 1,317 edges (*P* < 0.05).

Construction of a protein interaction network is a way to quickly analyse interactions between DEGs. We constructed a PPI network with 1,133 nodes and 11,112 edges using String and Cytoscape software (Figure 4A).

The Cytoscape plug-in MCODE was used to perform module analysis on the PPI network. We selected the two most meaningful modules for analysis and used DAVID to perform pathway enrichment analysis on the nodes of the module. Module 1 contains 73 nodes and 2,497 edges (Figure 4B). Through pathway enrichment analysis, DEGs of Model 1 were shown to be mainly enriched in cell cycle, p53 signaling pathway, progesterone-mediated oocyte maturation and oocyte meiosis signaling pathway. Module 2 contains 75 nodes and 1,317 edges (Figure 4C), and pathway enrichment analysis focuses on neuroactive ligand-receptor interaction, chemokine signaling pathway, and cytokine-cytokine receptor interaction signaling pathway.

Cytoscape's plug-in CytoNCA performs topology analysis on PPI networks. According to the results of the topological analysis based on comprehensive ranking of screening criteria, such as “degree,” the nodes with the best meaning in the network map were screened out, and the first ten DEGs were identified as targets for continued screening, including DNA topoisomerase 2-alpha (TOP2A), insulin (INS), interleukin-6 (IL6), mitotic-specific cyclin-B1 (CCNB1), histone H3-like centromeric protein A (CENPA), cyclin-A2 (CCNA2), aurora kinase B (AURKB), DNA repair protein RAD51 homolog 1 (RAD51), cell division control protein 6 homolog (CDC6), and polo-like kinase 1 (PLK1) (Table 1).

**Table 1:** The 10 most important DEGs identified from topology analysis of the PPI network.

**ID**	**Gene**	**Degree**	**Betweenness**	**Closeness**
1	TOP2A	174	73,188.05	0.033944048
2	INS	167	149,800.67	0.034301993
3	IL6	136	108,030.82	0.034162242
4	CCNB1	129	6,981.2573	0.0338183
5	CENPA	128	4,141.0425	0.033703517
6	CCNA2	122	7,042.7974	0.033711545
7	AURKB	122	6,464.02	0.033665426
8	RAD51	119	27,845.957	0.033836495
9	CDC6	119	4,479.0244	0.033593494
10	PLK1	118	12,405.141	0.03377088

### Survival Analysis

**Figure 5:**
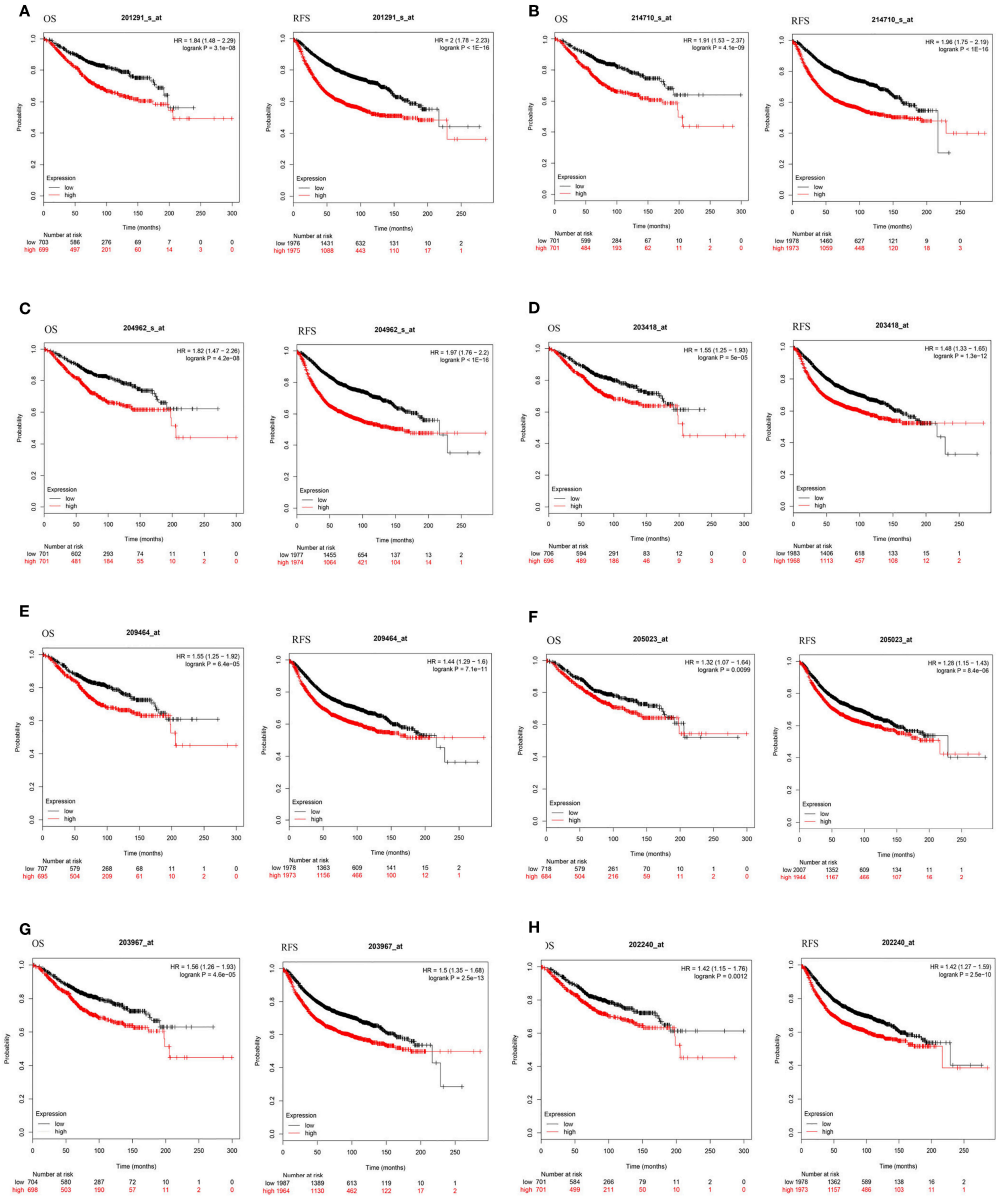
Kaplan-Meier curves of eight prognostic DEGs in BC. Notes: The order of eight prognostic DEGs are as follows: **(A)** TOP2A, **(B)** CCNB1, **(C)** CENPA, **(D)** CCNA2, **(E)** AURKB, **(F)** RAD51, **(G)** CDC6, and **(H)** PLK1. The criterion is that when the *P*-value of RFS and OS were <0.05, it is considered that DEGs are associated with the survival of BC.

To investigate the prognostic value of these 10 DEGs, the Kaplan-Meier plotter bioinformatics analysis platform was used. Analysis showed that from the 10 identified DEGs, only the INS and IL6 were not statistically relevant to the survival of BC. We found that high expression of eight DEGs was associated with unfavorable RFS and OS in BC patients, suggesting the remaining eight DEGs can be used as biomarkers for BC (Figure 5).

### Molecular Docking Model

**Figure 6:**
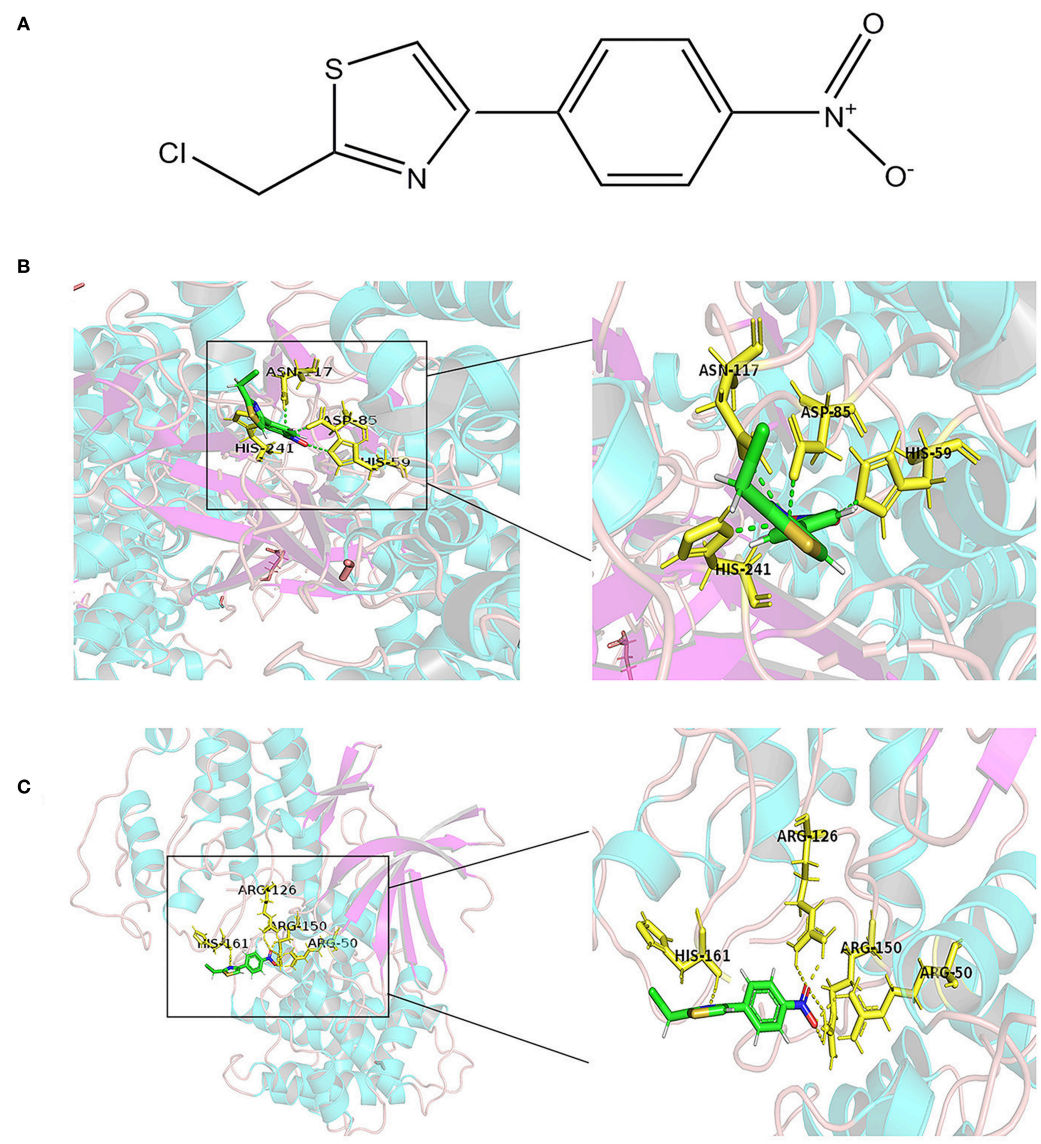
Docking combination of APS and key proteins. **(A)** Chemical structure of APS: 2-(Chloromethyl)-4-(4-nitrophenyl)-1,3-thiazole. The green structure is the *Astragalus* polysaccharide, and the yellow structure represents the binding site of CDC6 **(B)** and CCNB1 **(C)**.

Molecular docking is a theoretical simulation method for studying the interaction between molecules, such as ligands and receptors, and for predicting their binding mode and affinity. In recent years, molecular docking has become an important technology in the field of computer-aided drug research (26). In this study, to further explore the mechanism of interaction between APS and the eight DEGs, we constructed a molecular docking model of APS and the DEGs. We found that CCNB1 (PDB ID: 2JGZ, docking score: 5.2146) and CDC6 (PDB ID: 4I5N, docking score: 5.7514) have a stable binding site in the APS small molecule model, and the residues of APS interact with hydrogen bonds in the binding site (Figure 6).

### Analysis of Key Target Characteristics

**Figure 7:**
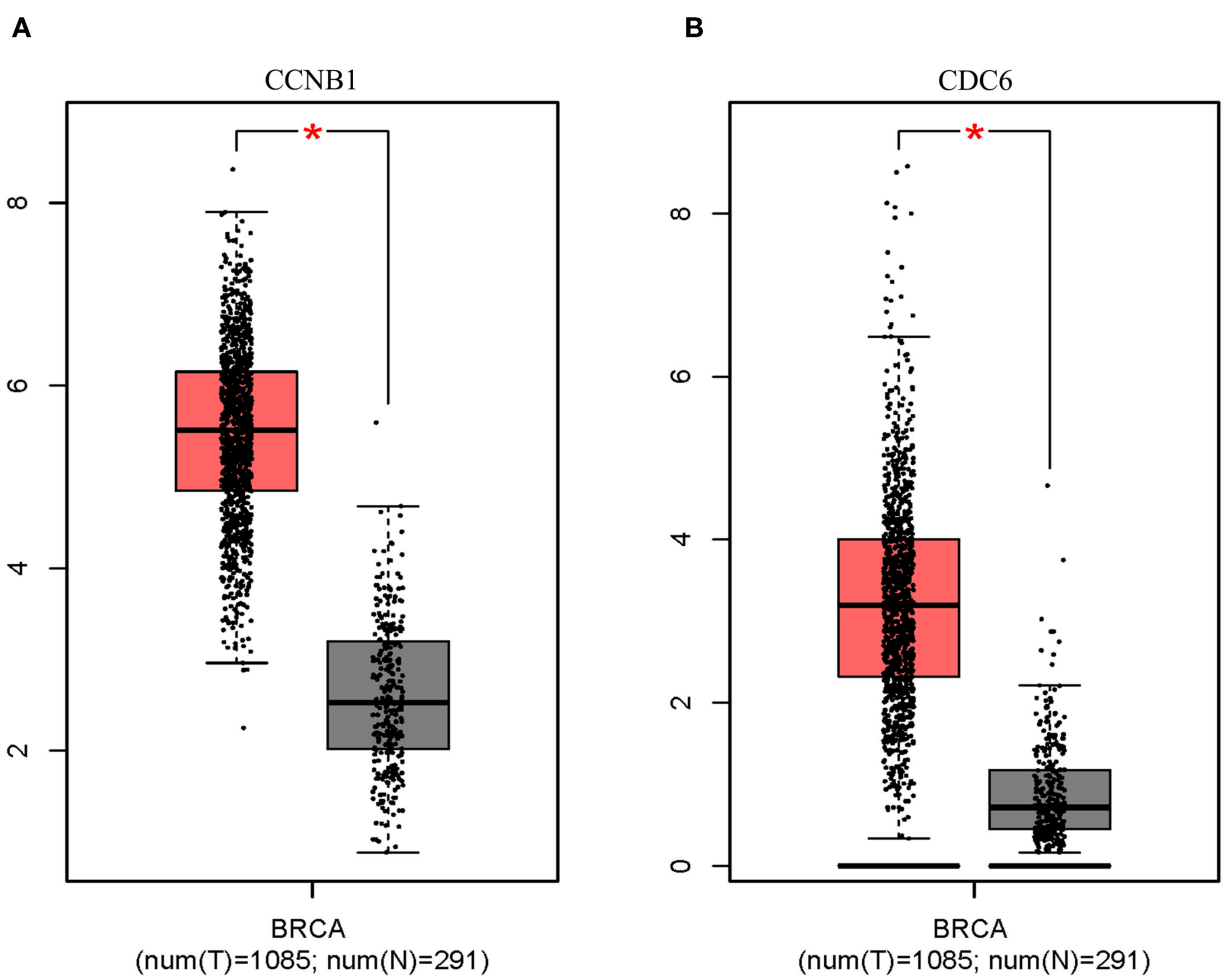
Expression on Box Plots of CCNB1 **(A)** and CDC6 **(B)**. Significant difference in expression was set to ^*^p < 0.01 vs. Normal sample.

**Figure 8:**
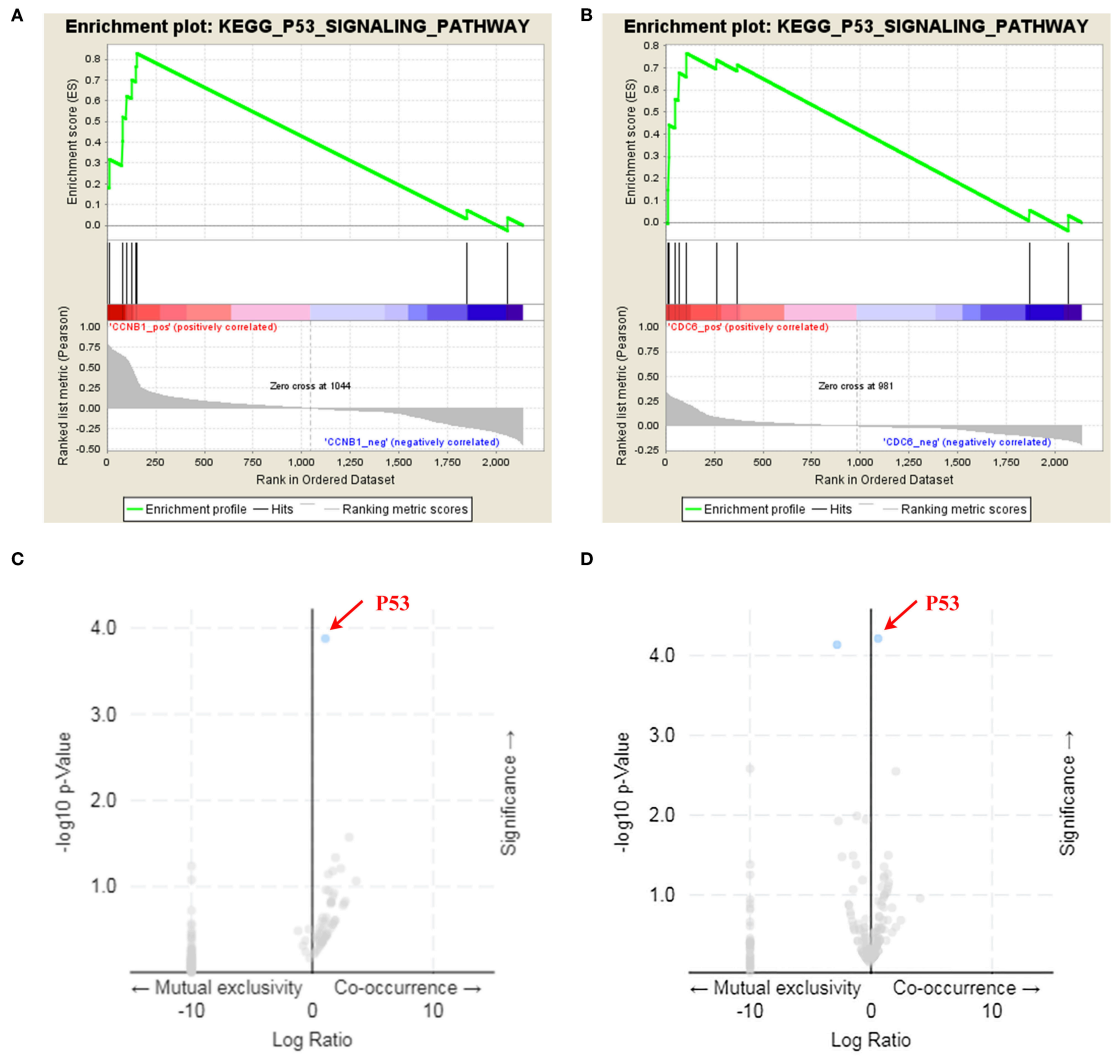
GSEA enrichment analysis and co-mutation volcano plot for CCNB1 and CDC6. **(A,B)** Identification of the enriched genesets with GSEA analysis focused on a single gene as a phenotype. Over-representation of positive (NES = 1.778, *p*-Value = 0.004) and CDC6 (NES = 1.469, *p*-Value = 0.05) was associated with P53 signaling pathway. **(C,D)** Both CCNB1 (*p*-Value = 1.323e-4, *q*-Value = 0.0229) and CDC6 (*p*-Value = 6.106e-5, *q*-Value = 6.331e-3) showed a co-mutation with P53.

Box plot showed a significant upregulation of CCNB1 and CDC6 in BC samples (Figure 7). Based on GSEA, we observed that the differential regulation of CCNB1 and CDC6 was significantly enriched in P53 signaling pathway (Figures 8A,B). And interestingly, both CCNB1 and CDC6 showed a significantly co-occurrence with P53 in mutation (Figures 8C,D). Therefore, key targets CCNB1, CDC6 and P53 were identified for experimental verification.

### Effect of APS on Cellular Viability

**Figure 9:**
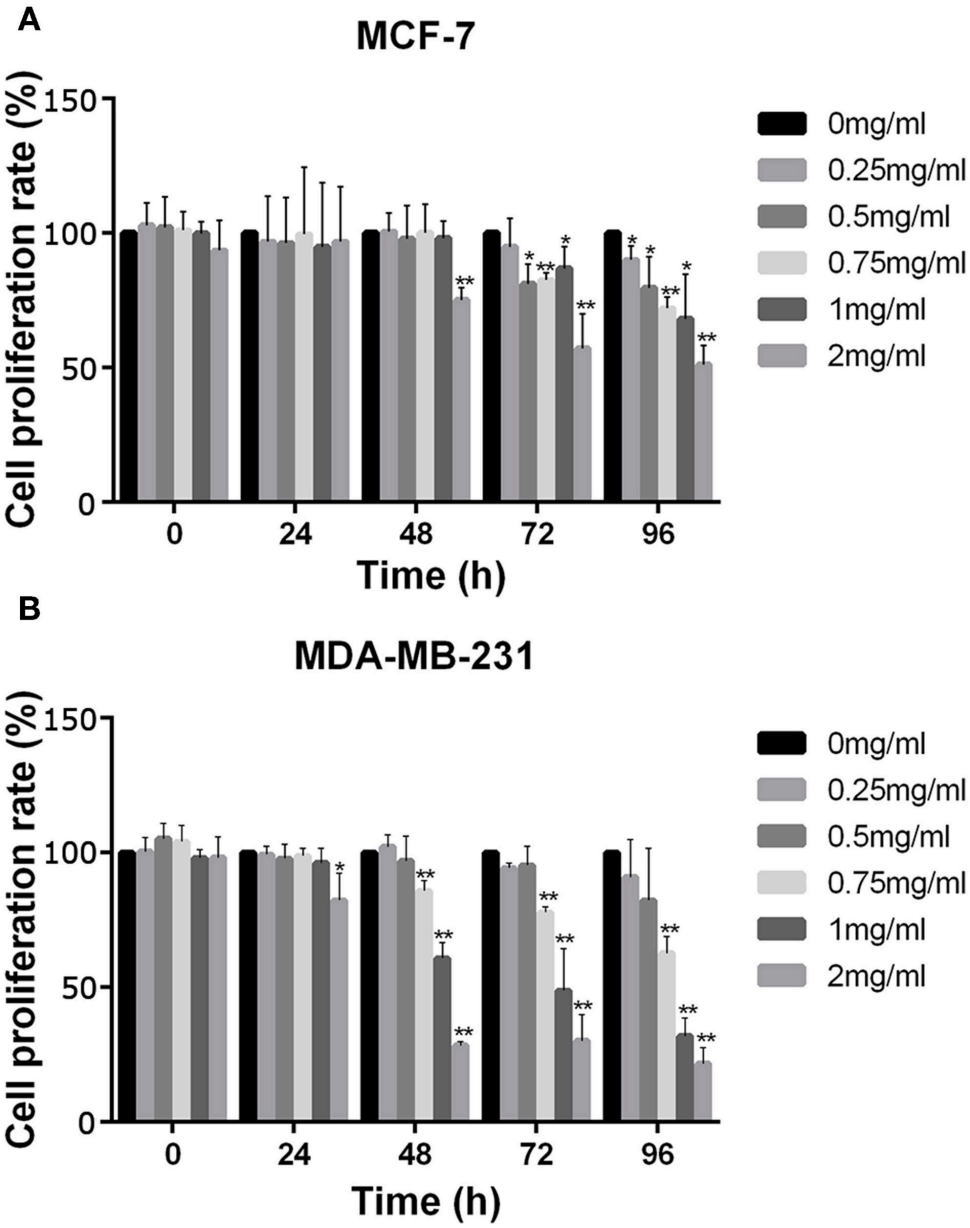
The increase in APS concentration significantly inhibited the activity of BC cells MCF-7 and MDA-MB-231. Optical density values at indicated concentrations of APS in MCF-7 **(A)** and MDA-MB-231 **(B)** cells were detected by CCK-8 assay, and absorbance was read at 450 nm. ^*^p < 0.05, ^**^p < 0.01 vs. control (0 mg/ml).

The effect of APS on cell viability of MCF-7 and MDA-MB-231 were measured using the Cell Counting Kit 8 (CCK-8) assay. As shown in Figure 9, the cell proliferation rate of MCF-7 decreased significantly after administered at all tested concentrations of APS over 72 h, while which of MDA-MB-231 decreased significantly after administered at all tested concentrations of APS over 48 h, although there was no significant inhibition of 0.25 and 0.5 mg/mL APS on MCF-7 and MDA-MB-231 cells when administered for 24 h. It suggested that APS could inhibited MCF-7 and MDA-MB-231 cells proliferation in a dose- and time-dependent manner.

### Cell Migration and Invasion Assays

**Figure 10:**
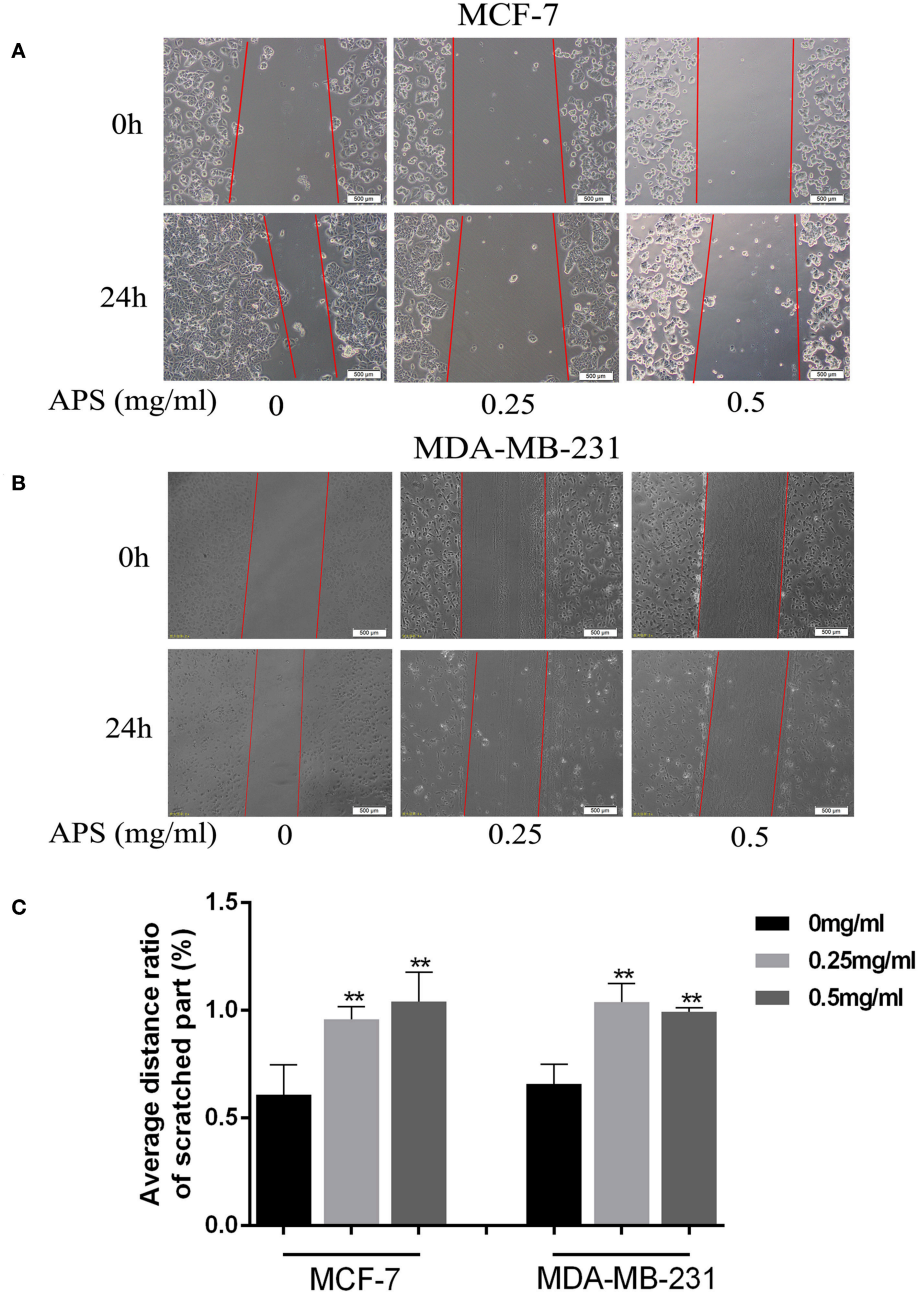
APS significantly inhibited cell migration of BC cells. Effects of APS (0, 0.25, 0.5 mg/ml) on migration of BC cells, MCF-7, and MDA-MB-231 cells were used to assess cell migration, the distance of cell movement was measured and photographed under an inverted microscope (100×). **(A,B)** Representative images of cells that migrated during wound-healing. **(C)** Graph of average distance ratio of scratches. ^*^p < 0.05, ^**^p < 0.01 vs. control (0 mg/ml).

**Figure 11:**
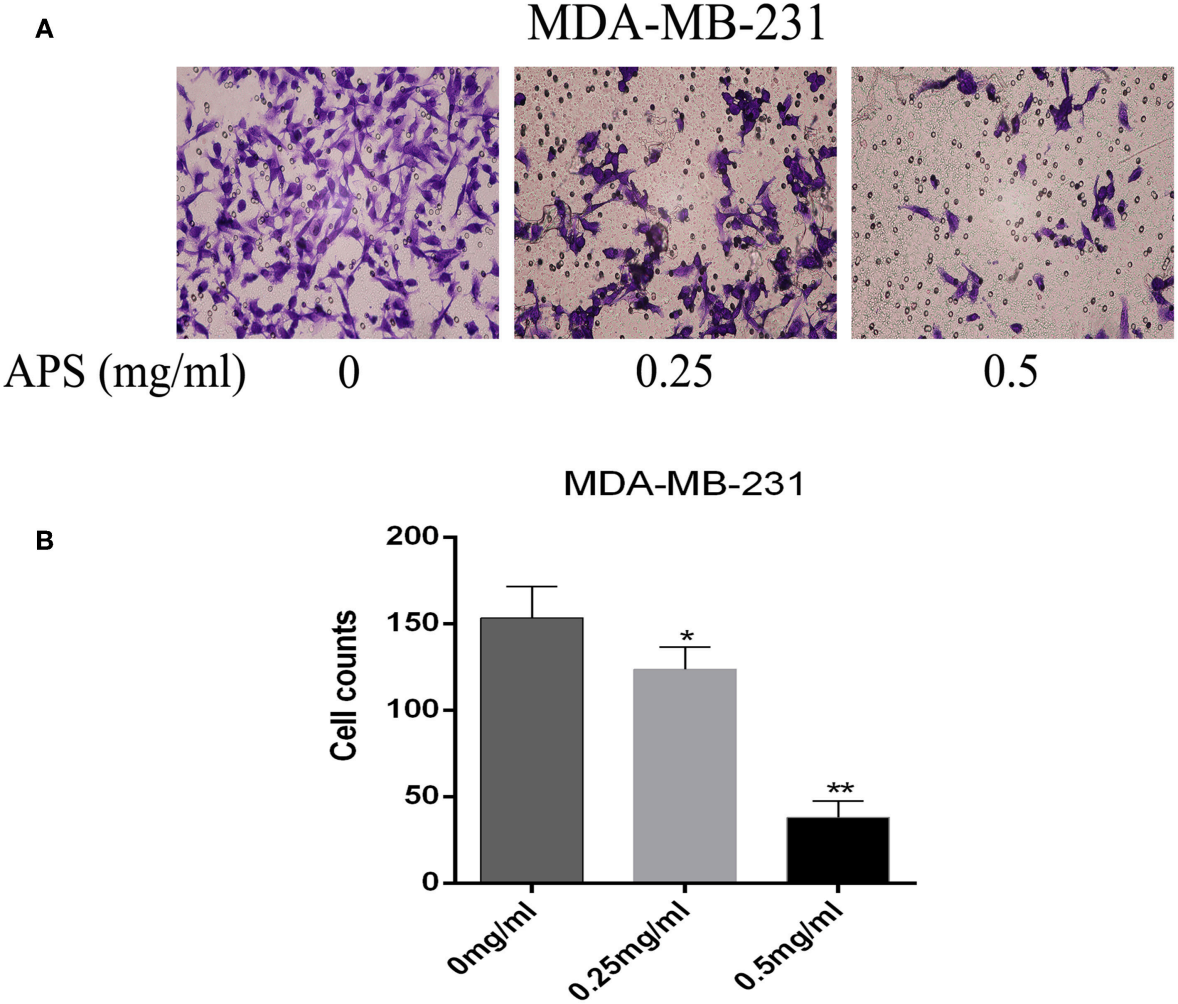
Dose-dependent inhibitory effects of APS on the invasion and motility of BC cell (MDA-MB-231) detected by Transwell assays. Cell motility of MDA-MB-231 cell in response to APS (0, 0.25, 0.5 mg/ml) treatment. **(A)** Representative micrographs of cell invasion. **(B)** Quantitative representation of cell numbers obtained from counting in three separate experiments. ^*^p < 0.05, ^**^p < 0.01 vs. control (0 mg/ml).

To further validate the effects of APS on breast cancer cell lines, cell migration and transwell invasion assays were performed. The results showed that the migration activity of MCF-7 and MDA-MB-231 cells was significantly inhibited by 0.25 and 0.5 mg/mL APS compared to control group (Figure 10). And the invasion activity of MDA-MB-231 cells with high invasive was significantly suppressed by 0.25 and 0.5 mg/mL APS compared to control group (Figure 11). It suggested that APS could inhibited the migration and invasion activity of human breast cancer cells.

### Expression of CCNB1, CDC6, and P53 in MDA-MB-231 Cells

**Figure 12:**
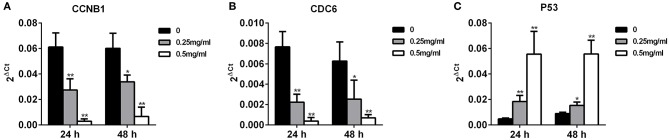
Increased APS dose can effectively reduce the expression of CCNB1 and CDC6 and increase the expression of P53. MDA-MB-231 cell was treated for 24 or 48 h with the indicated concentrations (0, 0.25, 0.5 mg/ml) of APS, levels of CCNB1 **(A)**, CDC6 **(B)**, and P53 **(C)** were determined using qRT-PCR. ^*^p < 0.05, ^**^p < 0.01 vs. control (0 mg/ml).

To compare the expression of CCNB1, CDC6, and P53 in invasive MDA-MB-231 cells with APS treatment, qRT-PCR were performed. As shown in Figure 12, after treated with 0.25 and 0.5 mg/mL APS, the expression of CCNB1 and CDC6 in MDA-MB-231 cells were inhibited significantly in a dose- and time-dependent manner, while the expression of P53 in MDA-MB-231 cells was concomitantly increased in a dose- and time-dependent manner. It indicated that APS could suppress the invasion of MDA-MB-231 cells by inhibiting the expression of CCNB1 and CDC6 and promoting the expression of P53, which was consistent with the results of bioinformatics analysis.

## Discussion

A malignant and complex systemic disease often consists of pathological alterations of polygene products and signaling pathways with potentially redundant or divergent functions (27). These pathways tend to be resilient in response to a single-target drug, resulting in difficulty correcting the disease state. One such disease, breast cancer, does not seem to show a very effective response to single treatment strategies. In this study, we constructed a multi-layered network to predict drug targets in a holistic manner using network pharmacological drug mechanism discovery methods, including screening gene-targets and identifying multiple targets. As this study has shown, the availability of high-throughput data and molecular networks provides an opportunity to study potential targets and drug intervention mechanisms for complex diseases.

The evolving method of network pharmacology provides such a paradigm, which aims at identifying compounds that regulate the activity of multiple targets in the impaired mechanisms network and deregulate interactions underlying a disease phenotype, either by targeting multiple pathways or by reducing potential adverse effects (28, 29). In such a network environment, therapeutic response can be taken into account from the robustness of complex disease networks to deal with node attacks, due to the inherent diversity and redundant compensation signaling pathways that result in highly resilient network systems with topological interactions (30). Specific intervention concepts for network targeting reveal the complex mechanisms of drug action, suggesting that the effects of small molecule compounds may have multifactorial interventions on different targets (31). This multi-target intervention model can lead to disturbances at different levels within biological networks, such as changes in gene expression or protein interactions (32). Multi-target effects are thought to overcome the adverse reactions associated with high doses of a single drug by resisting pathway compensation, thereby increasing therapeutic effects while minimizing overlapping toxicity (33). This is in line with the modern treatment concepts for complex diseases. Further, molecular docking is used to screen for affinity between drugs and targets, which can directly reveal the interaction between drug molecules and targets to clarify their structure-activity relationship (34, 35). This method exhibits great potential to provide preliminary hypotheses for previous *in vitro* and *in vivo* targeted validation studies.

Cancer chemotherapy places hope on the availability of drugs with ideally minimal toxicities and high levels of efficacy (36). For this reason, drugs developed from natural products may be viable options in cancer management. To live up to these expectations, natural products and their extracts must demonstrate their effectiveness as chemotherapeutic. A typical example is the mineral tetra-arsenic tetrasulfide (also known as Realgar) that has been used to treat human acute promyelocytic leukemia (37). Meanwhile, a range of plant-based active compounds, such as quercetin, curcumin, soy isoflavones, lentinan, etc. have been identified as potential chemopreventive agents (38–41). Moreover, natural product ingredients also demonstrated synergistic effects when used in combination with existing chemotherapeutic agents (42, 43).

APS, the main active ingredient extracted from the natural product Astragalus membranaceus, is a promising strategy for adjuvant treatment of cancer. It possesses a variety of immunomodulatory functions, synergism and reduced toxicity, including inhibiting growth and migration of CD4^+^CD25^+^Treg cells (44), enhancing the cellular activity of macrophages (45), increasing the sensitivity of cervical cancer HeLa cells and ovarian cancer SKOV3 cells to cisplatin (46, 47), etc. Recent research has focused on its anti-tumor activity for effective interventions in gastric cancer and liver cancer (48, 49). In our study, we used MCF-7 and MDA-MB-231 cells to study the effects of APS on breast cancer cells. Results illustrated that intervention with APS significantly inhibits the proliferation of breast cancer cells and reduces their migration and invasion. However, the mechanism of Astragalus polysaccharide intervention in breast cancer is still elusive. Our study utilized TCGA disease data and molecular target data through network pharmacology to demonstrate that Astragalus polysaccharide intervention in breast cancer may occur through regulation of CDC6 and CCNB1.

CDC6, a replication licensing factor, is responsible for loading mini-chromosome maintenance proteins into origin (50). Its deranged expression is not only a reflection of increased proliferation but also a necessary condition for initiating DNA replication as a potential “driving force” (51, 52). However, not until recently was the transcriptional regulation of CDC6 extensively linked to the development of cancer. CDC6 is frequently over-expressed in various epithelial cell carcinomas from early developmental stages (53). CCNB1 is a regulator of cell mitosis. As an essential cell cycle regulating factor, malfunctioning CCNB1 might be a proto-oncogene. It is often upregulated in a variety of human cancers (54–56), especially breast cancer (57). Our qRT-PCR data showed that with the increase of APS concentration and intervention duration, expression of CDC6 and CCNB1 was significantly decreased. In addition, the KEGG pathway assay showed that both CDC6 and CCNB1 were enriched in the p53 signaling pathway. P53 is the most commonly mutated gene in human cancer, and the inactivation of tp53-regulated pathways has been described in more than 50% of human cancers (58). Classical thinking supports that restoration of the p53 pathway may be an effective method for breast cancer treatment (59). In fact, previous studies have also shown that both CDC6 and CCNB1 are closely related to the P53 gene (Figure S1). They intervene with wild-type P53 to realize a synergistic effect on chromosome instability, cancer cell proliferation, and survival (53, 57). Therefore, we consider that P53 is also a key player in APS intervention in breast cancer cells, and the high expression of P53 observed by qRT-PCR after APS intervention confirmed this conclusion.

Our results reflect the potential pathological role of CCNB1, CDC6, and P53 in BC, as well as their being effective targets for APS intervention in breast cancer cells. The specific mechanism is that APS directly or indirectly affects the expression of CCNB1 and CDC6 by interfering with the upstream p53 gene, and finally realizes the regulation of breast cancer cells. Although our experiment is simple and lacks Positive drug control and normal breast cell control, the experimental results indirectly confirm the feasibility of our integrated approach to combining natural compounds with disease. This is important because, on the one hand, our experimental research explores the effective mechanism of APS intervention in the development of breast cancer; more importantly, we hope to construct such a comprehensive model. That is, the combination of network pharmacology and bioinformatics was used as preliminary prediction, and finally carried out experimental verification. The more important role of the experimental part is to verify the possibility of the target screening results, so as to explain the significance of our comprehensive model. These results can be used as a paradigm to effectively apply the mechanism of multi-target intervention of natural products with the advantages of improving efficiency and innovation, reducing clinical loss, and so on.

## Conclusion

In conclusion, our results indicate that expression of CCNB1 and CDC6 in breast cancer tissues is higher than in adjacent normal breast tissues. OS and RFS rates in patients with positive expression of both genes were significantly lower. APS can be considered an effective inhibitor of BC treatment by decreasing in cell proliferation, migration and invasion through regulating CCNB1, CDC6, and P53 and may be used as a supplement to BC therapy. More important, the present study also demonstrates the utility of Network Pharmacology in conjunction with TCGA database to discover the effectiveness of natural products in the intervention of cancer.

## Data Availability

Publicly available datasets were analyzed in this study. This data can be found here: https://cancergenome.nih.gov.

## Author Contributions

CL, HL, and CS conceptualized and designed this research. CG, JZ, and LL mined and analyzed original data. KW, FF, FC, and WZ performed the experiments. CL, HL, and CZ wrote the paper. CS and KW reviewed and edited the manuscript.

### Conflict of Interest Statement

The authors declare that the research was conducted in the absence of any commercial or financial relationships that could be construed as a potential conflict of interest.

## Supplementary Material

Figure S1 CCNB1 and CDC6 act as downstream genes of P53 and regulate DNA biosynthesis pathway and cell cycle arrest pathway.

## References

[1] Siegel RL Miller KD Jemal A Cancer statistics, 2017. CA Cancer J Clin. (2017) 67:7–30. 10.3322/caac.21387 28055103

[2] Cheng YJ Nie XY Ji CC Lin XX Liu LJ Chen XM. Long-term cardiovascular risk after radiotherapy in women with breast cancer. J Am Heart Assoc. (2017) 6:e005633. 10.1161/jaha.117.005633 28529208 PMC5524103

[3] Chen Y Zhang Y. Application of the CRISPR/Cas9 system to drug resistance in breast cancer. Adv Sci. (2018) 5:1700964. 10.1002/advs.201700964 29938175 PMC6010891

[4] Barabasi AL Gulbahce N Loscalzo J. Network medicine: a network-based approach to human disease. Nat Rev Genet. (2011) 12:56–68. 10.1038/nrg2918 21164525 PMC3140052

[5] Hassan AA The evolution of drug discovery: from phenotypes to targets, and back. MedChemComm. (2016) 7:788–98. 10.1039/C6MD00129G

[6] Hopkins AL. Network pharmacology: the next paradigm in drug discovery. Nat Chem Biol. (2008) 4:682–90. 10.1038/nchembio.118 18936753

[7] Tang J Aittokallio T. Network pharmacology strategies toward multi-target anticancer therapies: from computational models to experimental design principles. Curr Pharma Des. (2014) 20:23–36. 10.2174/13816128113199990470 23530504 PMC3894695

[8] Liu J Lichtenberg T Hoadley KA Poisson LM Lazar AJ Cherniack AD. An integrated TCGA pan-cancer clinical data resource to drive high-quality survival outcome analytics. Cell. (2018) 173:400–16.e11. 10.1016/j.cell.2018.02.052 29625055 PMC6066282

[9] Li X Pasche B Zhang W Chen K. Association of MUC16 mutation with tumor mutation load and outcomes in patients with gastric cancer. JAMA Oncol. (2018) 4:1691–8. 10.1001/jamaoncol.2018.2805 30098163 PMC6440715

[10] Hutter C Zenklusen JC. The cancer genome atlas: creating lasting value beyond its data. Cell. (2018) 173:283–5. 10.1016/j.cell.2018.03.042 29625045

[11] Leemans CR Snijders PJF Brakenhoff RH The molecular landscape of head and neck cancer. Nat Rev Cancer. (2018) 18:269–82. 10.1038/nrc.2018.11 29497144

[12] Cancer Genome Atlas Network Comprehensive molecular portraits of human breast tumours. Nature. (2012) 490:61–70. 10.1038/nature11412 23000897 PMC3465532

[13] Cancer Genome Atlas Network Genomic classification of cutaneous melanoma. Cell. (2015) 161:1681–96. 10.1016/j.cell.2015.05.044 26091043 PMC4580370

[14] Tomczak K Czerwinska P Wiznerowicz M. The Cancer Genome Atlas (TCGA): an immeasurable source of knowledge. Contemp Oncol. (2015) 19:A68–77. 10.5114/wo.2014.47136 25691825 PMC4322527

[15] Jiao X Sherman BT Huang da W Stephens R Baseler MW Lane HC. DAVID-WS: a stateful web service to facilitate gene/protein list analysis. Bioinformatics. (2012) 28:1805–6. 10.1093/bioinformatics/bts251 22543366 PMC3381967

[16] Su G Morris JH Demchak B Bader GD. Biological network exploration with Cytoscape 3. Curr Protoc Bioinform. (2014) 47:8.13.1–24. 10.1002/0471250953.bi0813s47 25199793 PMC4174321

[17] Tang Y Li M Wang J Pan Y Wu FX. CytoNCA: a cytoscape plugin for centrality analysis and evaluation of protein interaction networks. Bio Syst. (2015) 127:67–72. 10.1016/j.biosystems.2014.11.005 25451770

[18] Li D Zhao D Zhang W Ma Q Liu D Huang Q. Identification of proteins potentially associated with renal aging in rats. Aging. (2018) 10:1192–205. 10.18632/aging.101460 29907735 PMC6046247

[19] Xu H Bai X Yu S Liu Q Pestell RG Wu K. MAT1 correlates with molecular subtypes and predicts poor survival in breast cancer. Chinese J Cancer Res. (2018) 30:351–63. 10.21147/j.issn.1000-9604.2018.03.07 30046229 PMC6037588

[20] Wang Z Wang Y Zhang X Zhang T. Pretreatment prognostic nutritional index as a prognostic factor in lung cancer: review and meta-analysis. Clin Chim Acta. (2018) 486:303–10. 10.1016/j.cca.2018.08.030 30138620

[21] Jain AN. Surflex-Dock 2.1: robust performance from ligand energetic modeling, ring flexibility, and knowledge-based search. J Comp Aided Mol Des. (2007) 21:281–306. 10.1007/s10822-007-9114-2 17387436

[22] Cousins KR. Computer review of ChemDraw Ultra 12.0. J Am Chem Soc. (2011) 133:8388. 10.1021/ja204075s 21561109

[23] Jain AN. Surflex: fully automatic flexible molecular docking using a molecular similarity-based search engine. J Med Chem. (2003) 46:499–511. 10.1021/jm020406h 12570372

[24] Li X Ye L Wang X Wang X Liu H Zhu Y. Combined 3D-QSAR, molecular docking and molecular dynamics study on thyroid hormone activity of hydroxylated polybrominated diphenyl ethers to thyroid receptors beta. Toxicol Appl Pharmacol. (2012) 265:300–7. 10.1016/j.taap.2012.08.030 22982074

[25] Gao Y Qi W Sun L Lv J Qiu W Liu S. FOXO3 inhibits human gastric adenocarcinoma (AGS) cell growth by promoting autophagy in an acidic microenvironment. Cell Physiol Biochem. (2018) 49:335–48. 10.1159/000492884 30138933

[26] Fontana RJ Cirulli ET Gu J Kleiner D Ostrov D Phillips E. The role of HLA-A ^*^33:01 in patients with cholestatic hepatitis attributed to terbinafine. J Hepatol. (2018) 69:1317–25. 10.1016/j.jhep.2018.08.004 30138689 PMC6472700

[27] Borisy AA Elliott PJ Hurst NW Lee MS Lehar J Price ER. Systematic discovery of multicomponent therapeutics. Proc Natl Acad Sci USA. (2003) 100:7977–82. 10.1073/pnas.1337088100 12799470 PMC164698

[28] Kitano H. A robustness-based approach to systems-oriented drug design. Nat Rev Drug Discov. (2007) 6:202–10. 10.1038/nrd2195 17318209

[29] Lehar J Krueger AS Avery W Heilbut AM Johansen LM Price ER. Synergistic drug combinations tend to improve therapeutically relevant selectivity. Nat Biotechnol. (2009) 27:659–66. 10.1038/nbt.1549 19581876 PMC2708317

[30] Zhang C Zhou W Guan DG Wang YH Lu AP. Network intervention, a method to address complex therapeutic strategies. Front Pharmacol. (2018) 9:754. 10.3389/fphar.2018.00754 30050441 PMC6052041

[31] Shao L Network systems underlying traditional Chinese medicine syndrome and herb formula. Curr Bioinform. (2009) 4:188–96. 10.2174/157489309789071129

[32] Akker BVD Ibarz B Memmesheimer RM Self-organized criticality in a model for developing neural networks. BMC Neurosci. (2011) 12:P221 10.1186/1471-2202-12-S1-P221

[33] Buckley MM Sorkin EM. Enoxaparin. A review of its pharmacology and clinical applications in the prevention and treatment of thromboembolic disorders. Drugs. (1992) 44:465–97. 1382939 10.2165/00003495-199244030-00010

[34] Ferreira LG Dos Santos RN Oliva G Andricopulo AD. Molecular docking and structure-based drug design strategies. Molecules. (2015) 20:13384–421. 10.3390/molecules200713384 26205061 PMC6332083

[35] Vilar S Sobarzo-Sanchez E Santana L Uriarte E. Molecular docking and drug discovery in beta-adrenergic receptors. Curr Med Chem. (2017) 24:4340–59. 10.2174/0929867324666170724101448 28738772

[36] Balunas MJ Kinghorn AD. Drug discovery from medicinal plants. Life Sci. (2005) 78:431–41. 10.1016/j.lfs.2005.09.012 16198377

[37] Li XJ Zhang HY. Synergy in natural medicines: implications for drug discovery. Trends Pharmacol Sci. (2008) 29:331–2. 10.1016/j.tips.2008.04.002 18502520

[38] Qian Y Wang D Fan M Xu Y Sun X Wang J. Effects of intrinsic metal ions of lentinan with different molecular weights from Lentinus edodes on the antioxidant capacity and activity against proliferation of cancer cells. Int J Biol Macromolecules. (2018) 120:73–81. 10.1016/j.ijbiomac.2018.06.203 29981326

[39] Rauf A Imran M Khan IA Ur-Rehman M Gilani SA Mehmood Z. Anticancer potential of quercetin: a comprehensive review. Phytother Res. (2018) 32:2109–30. 10.1002/ptr.6155 30039547

[40] Hamzehzadeh L Atkin SL Majeed M Butler AE Sahebkar A. The versatile role of curcumin in cancer prevention and treatment: a focus on PI3K/AKT pathway. J Cell Physiol. (2018) 233:6530–7. 10.1002/jcp.26620 29693253

[41] Pudenz M Roth K Gerhauser C. Impact of soy isoflavones on the epigenome in cancer prevention. Nutrients. (2014) 6:4218–72. 10.3390/nu6104218 25322458 PMC4210915

[42] Kuo YL Chen CH Chuang TH Hua WK Lin WJ Hsu WH. Gene expression profiling and pathway network analysis predicts a novel antitumor function for a botanical-derived drug, PG2. Evid Based Complement Altern Med. (2015) 2015:917345. 10.1155/2015/917345 25972907 PMC4417974

[43] Li J Bao Y Lam W Li W Lu F Zhu X. Immunoregulatory and anti-tumor effects of polysaccharopeptide and Astragalus polysaccharides on tumor-bearing mice. Immunopharmacol Immunotoxicol. (2008) 30:771–82. 10.1080/08923970802279183 18686097

[44] Li Q Bao JM Li XL Zhang T Shen XH Inhibiting effect of Astragalus polysaccharides on the functions of CD4^+^CD25 high Treg cells in the tumor microenvironment of human hepatocellular carcinoma. Chinese Med J. (2012) 125:786–93. 10.3760/cma.j.issn.0366-6999.2012.05.012 22490576

[45] Cho WC Leung KN. *In vitro* and *in vivo* anti-tumor effects of Astragalus membranaceus. Cancer Lett. (2007) 252:43–54. 10.1016/j.canlet.2006.12.001 17223259

[46] Zhai QL Hu XD Xiao J Yu DQ. Astragalus polysaccharide may increase sensitivity of cervical cancer HeLa cells to cisplatin by regulating cell autophagy. Zhongguo Zhong Yao Za Zhi. (2018) 43:805–12. 10.19540/j.cnki.cjcmm.20171113.018 29600659

[47] Li C Hong L Liu C Min J Hu M Guo W. Astragalus polysaccharides increase the sensitivity of SKOV3 cells to cisplatin. Arch Gynecol Obstet. (2018) 297:381–6. 10.1007/s00404-017-4580-9 29103194

[48] Lai X Xia W Wei J Ding X. Therapeutic effect of astragalus polysaccharides on hepatocellular carcinoma H22-bearing mice. Dose Response. (2017) 15:1559325816685182. 10.1177/1559325816685182 28210201 PMC5298564

[49] Zhang D Zheng J Ni M Wu J Wang K Duan X. Comparative efficacy and safety of Chinese herbal injections combined with the FOLFOX regimen for treating gastric cancer in China: a network meta-analysis. Oncotarget. (2017) 8:68873–89. 10.18632/oncotarget.20320 28978164 PMC5620304

[50] Donovan S Harwood J Drury LS Diffley JF. Cdc6p-dependent loading of Mcm proteins onto pre-replicative chromatin in budding yeast. Proc Natl Acad Sci USA. (1997) 94:5611–6. 9159120 10.1073/pnas.94.11.5611 PMC20826

[51] Bartkova J Rezaei N Liontos M Karakaidos P Kletsas D Issaeva N. Oncogene-induced senescence is part of the tumorigenesis barrier imposed by DNA damage checkpoints. Nature. (2006) 444:633–7. 10.1038/nature05268 17136093

[52] Petrakis TG Komseli ES Papaioannou M Vougas K Polyzos A Myrianthopoulos V. Exploring and exploiting the systemic effects of deregulated replication licensing. Semin Cancer Biol. (2016) 37–38:3–15. 10.1016/j.semcancer.2015.12.002 26707000

[53] Komseli ES Pateras IS Krejsgaard T Stawiski K Rizou SV Polyzos A. A prototypical non-malignant epithelial model to study genome dynamics and concurrently monitor micro-RNAs and proteins *in situ* during oncogene-induced senescence. BMC Genomics. (2018) 19:37. 10.1186/s12864-017-4375-1 29321003 PMC5763532

[54] Wang Q Moyret-Lalle C Couzon F Surbiguet-Clippe C Saurin JC Lorca T. Alterations of anaphase-promoting complex genes in human colon cancer cells. Oncogene. (2003) 22:1486–90. 10.1038/sj.onc.1206224 12629511

[55] Fang Y Yu H Liang X Xu J Cai X. Chk1-induced CCNB1 overexpression promotes cell proliferation and tumor growth in human colorectal cancer. Cancer Biol Ther. (2014) 15:1268–79. 10.4161/cbt.29691 24971465 PMC4128869

[56] Kim SK Roh YG Park K Kang TH Kim WJ Lee JS. Expression signature defined by FOXM1-CCNB1 activation predicts disease recurrence in non-muscle-invasive bladder cancer. Clin Cancer Res. (2014) 20:3233–43. 10.1158/1078-0432.ccr-13-2761 24714775

[57] Fang L Du WW Lyu J Dong J Zhang C Yang W. Enhanced breast cancer progression by mutant p53 is inhibited by the circular RNA circ-Ccnb1. Cell Death Differ. (2018) 25:2195–208. 10.1038/s41418-018-0115-6 29795334 PMC6261950

[58] Hamroun D Kato S Ishioka C Claustres M Beroud C Soussi T. The UMD TP53 database and website: update and revisions. Hum Mutat. (2006) 27:14–20. 10.1002/humu.20269 16278824

[59] Moulder DE Hatoum D Tay E Lin Y McGowan EM. The roles of p53 in mitochondrial dynamics and cancer metabolism: the pendulum between survival and death in breast cancer? Cancers. (2018) 10:E189. 10.3390/cancers10060189 29890631 PMC6024909

