# Supplementary figures and images for: Identifying the Antiproliferative Effect of Astragalus Polysaccharides on Breast Cancer: Coupling Network Pharmacology With Targetable Screening From the Cancer Genome Atlas
Source: Front Oncol. 2019 May 17;9:368. doi: 10.3389/fonc.2019.00368 (PMC6533882; doi:10.3389/fonc.2019.00368)

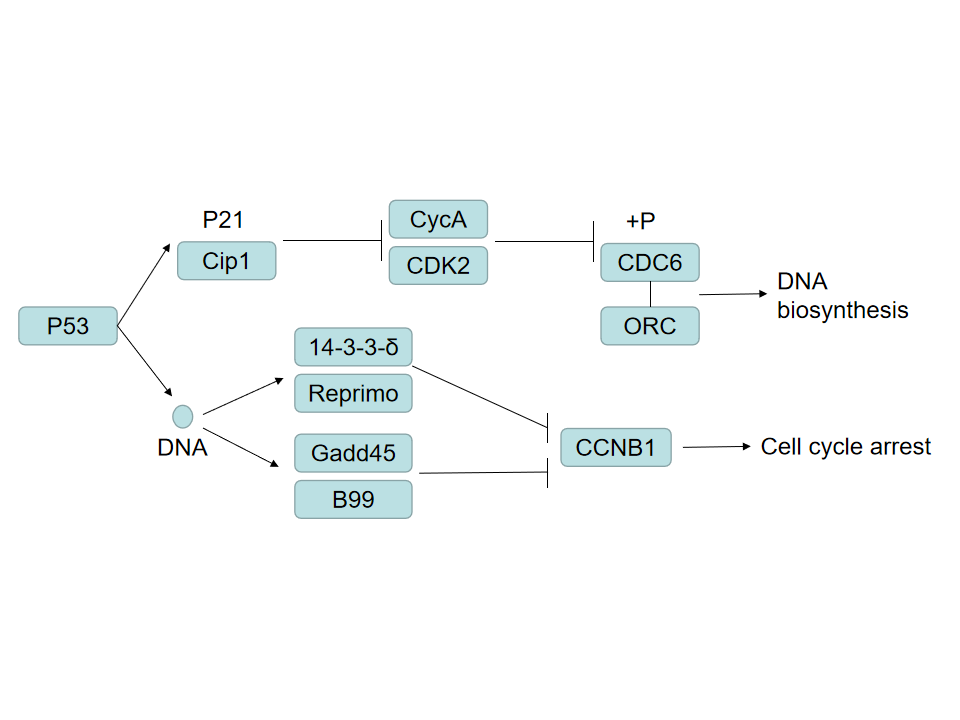

Supplement: Figure S1 — CCNB1 and CDC6 act as downstream genes of P53 and regulate DNA biosynthesis pathway and cell cycle arrest pathway. [file Image_1.TIF]
